# EXIT procedure in twin pregnancy: a series of three cases from a single center

**DOI:** 10.1186/1471-2393-14-252

**Published:** 2014-07-30

**Authors:** Lutgardo García-Díaz, Juan Carlos de Agustín, Antonio Ontanilla, Maria Luisa Marenco, Antonio Pavón, Antonio Losada, Guillermo Antiñolo

**Affiliations:** Unidad de Gestión Clínica de Genética, Reproducción y Medicina Fetal, Instituto de Biomedicina de Sevilla (IBIS), Hospital Universitario Virgen del Rocío/CSIC/Universidad de Sevilla, 41003 Sevilla, Spain; Unidad de Gestión Clínica de Cirugía Infantil, Hospital Universitario Virgen del Rocío, 41013 Sevilla, Spain; Servicio de Anestesiología Hospital Infantil, Hospitales Universitarios Virgen Del Rocío, 41013 Sevilla, Spain; Unidad De Gestión Clínica De Neonatología, Hospital Universitario Virgen Del Rocío, 41013 Sevilla, Spain; Servicio de Anestesiología Hospital de La Mujer, Hospital Universitario Virgen Del Rocío, 41013 Sevilla, Spain; Centro de Investigación Biomédica en Red de Enfermedades Raras (CIBERER), Sevilla, Spain

**Keywords:** Prenatal diagnosis, Fetal medicine, Fetal intervention, Twin pregnancy, Ex utero intrapartum therapy (EXIT) procedure

## Abstract

**Background:**

Indications for the ex utero intrapartum therapy (EXIT) procedure have evolved and nowadays in addition to secure the airway, obtain vascular access, administer surfactant and other resuscitation medications, EXIT is used to resect cervical or thoracic masses, for extracorporeal membrane circulation (ECMO) cannulation, as well as to rescue maximum intra-thoracic space for ventilation of the remaining functional lung tissue or in cases in which resuscitation of the neonate may be compromised. EXIT procedure in twin pregnancy has been rarely reported and some doubts have been raised about its strategy and safety in such cases.

**Methods:**

We reviewed the medical records of 3 twin pregnancy cases where the EXIT procedure have been performed in our center.

**Results:**

The mean gestational age at EXIT procedure was 34 + 4 weeks. In two out the three EXIT procedures, the affected twin was delivered first. The average time on placental bypass was 9 minutes. There were no fetal or maternal complications related to the EXIT procedure. All newborns are currently doing well.

**Conclusion:**

In twin pregnancies, prenatal diagnosis combined with the EXIT procedure permits the formulation of a controlled delivery strategy to secure both newborns outcome. In those pregnancies, if intervention can be accomplished without compromise of the normal twin, EXIT can be considered. Our results support that EXIT procedure, if properly planned, safely provides a good outcome for both the fetuses as well as the mother.

## Background

The ex-utero intrapartum therapy (EXIT) procedure was initially described for the reversal of tracheal occlusion at the time of delivery in fetuses with severe congenital diaphragmatic hernia (CDH) that had undergone in-utero tracheal occlusion [1, 2]. EXIT allows therapeutic interventions on the fetus while maintaining fetoplacental circulation and thereby maintaining oxygenation. In cases of airway obstruction, the EXIT procedure has proved to be very useful because it avoids the hypoxia, brain injury, and death that can be associated with a neonatal airway crisis. The time provided by the EXIT procedure converts an emergent crisis into a controlled situation. Over time, the techniques used in the EXIT procedure have become standardized, enabling surgeons to perform many differente procedures under controlled conditions, while the fetus remains on uteroplacental bypass. Progressively, the EXIT procedure indications have evolved and nowadays in addition to secure the airway, obtain vascular access, administer surfactant and other resuscitation medications, EXIT is used to resect cervical or thoracic masses, for extracorporeal membrane circulation (ECMO) cannulation, as well as to rescue maximum intra-thoracic space for ventilation of the remaining functional lung tissue [3–6].

In twin pregnancies, prenatal diagnosis combined with EXIT permits the formulation of a controlled delivery strategy to secure both newborns outcome. In those pregnancies, if intervention can be accomplished without compromise of the normal twin, EXIT can be considered. EXIT procedure in twin pregnancy has been rarely reported [7–10] and some doubts have been raised about its strategy and safety in twin pregnancies. In this paper we report our experience with the EXIT procedure in a series of three twin pregnancies, to our knowledge the largest series from a single center. The results are summarized in Table 1.

**Table 1:** **Results of the EXIT procedures in twin pregnancies**

	CASE 1	CASE 2	CASE 3
**DIAGNOSIS**	Giant epignathus	Congenital diaphragmatic hernia.	Congenital diaphragmatic hernia.
**AFFECTED TWIN**	Twin B	Twin A	Twin A
**GESTATIONAL AGE AT EXIT (WK)**	34	34 + 4	35
**TIME ON UTEROPLACENTAL CIRCULATION (MIN)**	7	7	14
**EXIT PROCEDURE**	Resection and tracheostomy	Airway intubation and surfactant administration	Airway intubation and surfactant administration
**NEONATAL HOSPITAL STAY (DAYS)**	78	61	22
**PREOPERATIVE MATERNAL HEMOGlOBIN (g/L)**	8.3	11.9	10.3
**POSTOPERATIVE MATERNAL HEMOGLOBIN (g/L)**	6.8	11.3	9.7
**POSTPARTUM HOSPITAL STAY (DAYS)**	4	4	4
**OUTCOME**	Both twins doing well 7 years after birth	Both twins doing well 5 years after birth	Both twins doing well 2 years after birth

## Methods

### Exit procedure

We used a combined technique, general plus epidural anesthesia, to ensure mother and fetus anesthesia during the exit procedures. An epidural catheter is placed to control intraoperative and postoperative pain. General anesthesia induction (remifentanilo, propofol and rocuronio) is followed in rapid sequence by intubation and assisted ventilation. Before the uterine incision deep inhalational anesthesia with sevoflurane is used to maintain uterine relaxation and preserve uteroplacental circulation and fetal gas exchange. A low transverse abdominal incision exposes the uterus, and a sterile intraoperative ultrasound probe maps the position of the placenta and confirms the position of the fetuses. The location of the hysterotomy is determined by the placental locations, and a margin of at least 5 cm from the lower placental edge is left. We use our uterine progressive distractors and a stapling device (Premium Poly Cs-57 Autosuture®), to enter into the amniotic sac with minimum uterine bleeding [11]. Fetuses are continuously monitored with fetal echocardiography. After delivery of the second fetus the closure of the uterus and the abdominal wall is performed according to the standard procedure described elsewhere.

## Results

An informed consent was obtained from all the patients for clinical studies. The study and procedures conformed to the tenets of the declaration of Helsinki and were approved by the Institutional Review Boards from the University Hospital Virgen del Rocío of Seville. Expressed consent was signed by all the cases for the publication of their clinical data and images if required.

### Case 1

A 33-year-old, gravida 1, para 0, at 21 weeks’ gestation, was referred to for evaluation of an abnormal twin gestation with a facial tumour in one of the fetuses. 2-D, 3-D and 4-D ultrasound showed a bicorionic, biamniotic twin pregnancy with a normal female twin A, and a female twin B with a large asymmetric anterior mass protruding from mouth and nose not affecting the neck area, occluding completely fetal airway and compromising perinatal transition to pulmonary breath at birth. Prenatal diagnosis of epignatus was established. MRI confirmed an epignatus not invading CNS. Once diagnose was established, our *Fetal Medicine and Therapy Program* multidisciplinary team proposed the parents to perform an ex utero intrapartum treatment procedure to secure the airway through tracheostomy and intubation before delivery. Implications, benefits and risk of the procedure as well as posterior neonatal treatment were discussed in detail. The parents decided to continue the pregnancy and accepted EXIT. Regular ultrasound assessments showed growth of the tumour to a maximum of 93 x 62 x 56 mm. Polyhydramnios developed in the affected twin as soon as 26 weeks’s gestation, resulting in uterine irritability, and requiring 3 amnioreduction procedures (at 27 and 30 weeks’ gestation and preoperative), tocolytic agents and bed rest. Dexamethasone injections were given at 27 weeks of gestation. The mother developed anaemia from 27 weeks’ gestation that reduced haemoglobin levels to 83 g/L and was treated with ferrous sulphate, 300 mg/day. The woman was admitted at 33 + 4 weeks of gestation for a planned delivery due to the high risk of early spontaneous onset of labour and premature rupture of membranes. At 34th week, the patient underwent the EXIT procedure. Twin A was delivered and passed to the neonatal resuscitation team. Due to the breech fetal position, twin B was completely delivered and positioned approximately at placental level. According to prenatal diagnosis, a large asymmetric mass protruding from mouth and nose, occluding completely the foetal airway was confirmed. Surgical excision of the extraoral mass using two fires of a surgical stapling device followed by tracheostomy were performed. Fetal airway was established with a 3-mm endotracheal tube. Then, the umbilical cord was clamped and divided, and twin B was passed to the neonatal resuscitation team. The time from mass excision and fetal tracheostomy and intubation to cord clamping was 7 minutes. The following day, trachea was repaired reversing the tracheostomy. Airway was secured using fiberoptic guided transnasal intubation. Eight days after delivery the patient underwent surgical revision obtaining complete resection of the tumor with ligation of the superior pharyngeal stalk. The newborn was discharged 78 days after birth. The mother was discharged on the fourth day after intervention. Both newborns are currently doing well.

### Case 2

A 33-year-old woman, gravid 2, para 1, with a bichorionic biamniotic twin pregnancy was referred to at 29 weeks’ gestation for evaluation of a left-sided congenital diaphragmatic hernia (CDH). Ultrasound showed a normal male fetus B, and a male fetus A with a left-sided CDH which included a portion of the liver. The lung-to-head ratio (LHR) was 1.2 an Observed/Expected (O/E) LHR was 28%. At 34 + 4 weeks’ gestation, an EXIT to ECMO procedure was performed. Fetus A was delivered first, airway was securely intubated and surfactant was administered during EXIT (7 minutes), afterwards the fetus B was delivered without problems. Birth’s weight were 2443 g (twin A) and 2260 g (twin B) respectively. ECMO was not needed and surgical correction of CDH was performed 48 hours later without complications. Newborn was discharged 61 days after surgical repair. The mother was discharged on the fourth day after intervention. Both newborns are currently doing well.

### Case 3

A 33-year-old woman, gravida 1, with a bichorionic biamniotic twin pregnancy, was referred to at 27 week’s because a CDH in one of the twins complicated by preterm labor. Ultrasound showed a normal male fetus B, and a male fetus A with the left hemithorax ocupated by stomach, small intestine and the left portion of the liver. LHR was 1.28 and O/E LHR was 28%. Tocolysis was started and betamethasone was administered for lung maturation. MRI confirmed a left sided CDH with liver up. EXIT to ECMO procedure was performed at 35 weeks gestation. Fetus A was delivered first, airway was securely intubated and surfactant was administered during EXIT (14 minutes), afterwards the second fetus was delivered without problems. The weight of the newborn with CDH was 2352 g. Surgical correction was performed without complications 24 hours later. Newborn was discharged 21 days after surgical repair. The mother was discharged on the fourth day after intervention. Both newborns are currently doing well.

## Discussion

Over the past 25 years, fetal surgery has evolved into a multidisciplinary specialty capable of improving perinatal outcome for a wide variety of diseases. Recent advances allow to accurately diagnose as well as treat a number of fetal anomalies while preserving maternal safety. The extensive use of prenatal ultrasound has resulted in the increased identification of a number of fetal disorders that have a direct impact in the perinatal management of the fetus and the subsequent outcome. In the last few years, the use of the EXIT procedure has been further expanded to include fetal anomalies where neonatal resuscitation may be compromised, including large thoracic lesions, bilateral hidrothorax, CDH, unilateral pulmonary agenesis, and cardiac lesions [4–7].

Twin pregnancy increases EXIT procedure complexity. In those cases, the control of key elements of an EXIT procedure requires an extreme coordination between all the members of the multidisciplinary team.

When using the EXIT procedure in a twin gestation, the risk should be borne by the affected twin. Priority should always be given to the normal twin in case of risk or complication. However, priority does not mean to necessarily deliver the normal twin first. In our experience, adequate uterine relaxation and careful monitoring of both fetuses and the mother, as well as a good coordination of the team allows treatment of the affected twin before delivery of the unaffected one without risk. Planning and coordination among the multidisciplinary team members during each phase of the EXIT procedure is the key for a successful outcome in those cases.

## Conclusions

In conclusion, in our series EXIT procedure in twin pregnancy have had very good results. Those results support that in twin pregnancy EXIT procedure, if properly planed, safely provides a good outcome for both the fetuses as well as the mother.
